# Weighted minimum feedback vertex sets and implementation in human cancer genes detection

**DOI:** 10.1186/s12859-021-04062-2

**Published:** 2021-03-22

**Authors:** Ruiming Li, Chun-Yu Lin, Wei-Feng Guo, Tatsuya Akutsu

**Affiliations:** 1 Bioinformatics Center, Institute for Chemical Research, Kyoto University, Uji, Kyoto 611-0011 Japan; 2 Institute of Bioinformatics and Systems Biology, National Yang Ming Chiao Tung University, 300 Hsinchu, Taiwan; 3 Center for Intelligent Drug Systems and Smart Bio-devices, National Yang Ming Chiao Tung University, 300 Hsinchu, Taiwan; 4 School of Electrical Engineering, Zhengzhou University, 450001 Zhengzhou, China

**Keywords:** Feedback vertex set, Differential gene expression, Cancer gene

## Abstract

**Background:**

Recently, many computational methods have been proposed to predict cancer genes. One typical kind of method is to find the differentially expressed genes between tumour and normal samples. However, there are also some genes, for example, ‘dark’ genes, that play important roles at the network level but are difficult to find by traditional differential gene expression analysis. In addition, network controllability methods, such as the minimum feedback vertex set (MFVS) method, have been used frequently in cancer gene prediction. However, the weights of vertices (or genes) are ignored in the traditional MFVS methods, leading to difficulty in finding the optimal solution because of the existence of many possible MFVSs.

**Results:**

Here, we introduce a novel method, called weighted MFVS (WMFVS), which integrates the gene differential expression value with MFVS to select the maximum-weighted MFVS from all possible MFVSs in a protein interaction network. Our experimental results show that WMFVS achieves better performance than using traditional bio-data or network-data analyses alone.

**Conclusion:**

This method balances the advantage of differential gene expression analyses and network analyses, improves the low accuracy of differential gene expression analyses and decreases the instability of pure network analyses. Furthermore, WMFVS can be easily applied to various kinds of networks, providing a useful framework for data analysis and prediction.

**Supplementary Information:**

The online version supplementary material available at 10.1186/s12859-021-04062-2.

## Background

Cancer is a genetic disease, but not all genes are related to cancer. By almost universal consensus, cancer is now viewed as resulting from changes in some key regulatory genes [1]. At present, researchers have defined several kinds of cancer-related gene sets. One widely used kind of gene set is that of cancer driver genes, which are defined as genes whose mutations increase net cell growth under the specific microenvironmental conditions that exist in the cell in vivo. This kind of gene can be predicted by finding ‘significantly mutated genes’, whose mutation rates are significantly higher than the presumed background somatic mutation rate  [2–4]. However, since it is difficult to construct a reliable background mutation model [5], selecting gold-standard driver genes by frequency-based methods is difficult. Another kind of cancer-related genes are so-called ‘cancer genes’, including oncogenes, which function as positive growth regulators, and tumour suppressor genes (TSGs), which function as negative growth regulators. These genes are directly related to the phenotypes of tumour and normal genes and can be predicted by differential gene expression analyses. However, some ‘dark’ genes play important roles at the network level but are generally ignored by traditional differential gene expression analyses [6, 7].

By using graph theory algorithms, we can find critical vertices to control a network. For example, [8] developed a feedback-based framework that provides realizable node overrides that steer a system towards one of its natural long-term dynamic behaviours; [9] provided a rational criterion for selecting key molecules to control a system with a feedback vertex set (FVS); [10] proposed a network control strategy to find driver mutations that drive a regulation network from the normal state to a disease state; and [11] considered applying minimum feedback vertex sets (MFVS) to real biologically directed complex networks and found essential proteins in both *Drosophila melanogaster* and *Homo sapiens* organisms.

Given a directed network, a feedback vertex set (FVS) is a set of vertices whose removal leaves the remaining network acyclic. The minimum feedback vertex set (MFVS) is a kind of FVS that has the minimum size among all possible FVSs. The MFVS problem has been proven to be NP-complete [12]. There already exist many algorithms for solving the MFVS problem, including approximation algorithms [13], randomized algorithms [14], parameterized algorithms [15] and exact algorithms [16, 17].

Generally, a network can have multiple MFVSs. Traditional MFVS algorithms ignore the differences among possible MFVSs, and the output is usually random. This randomness leads to the instability of network analysis methods in practice. To find the best output from multiple MFVSs, in this paper, we consider a variation of the MFVS problem, i.e., each vertex is assigned a weight, and the output is the maximum total weighted MFVS. The assigned weight should reflect the significance of the corresponding vertex, which may involve some biological data from other studies (for example, in our experiments, we utilize the differential expression value to compute the weights). We define this problem as a weighted MFVS (WMFVS) problem.

To solve the WMFVS problem, we modified an exact algorithm from [17], which first compresses the original graph [18, 19] to reduce the number of vertices and arcs and then utilizes an integer linear programming (ILP) method for the compressed graph. Our WMFVS method can be roughly separated into three parts, i.e., graph compression, MFVS size determination and output optimization. The first two parts use the same idea as [17], and the third part uses the modified ILP method to select the maximum weighted MFVS.

Furthermore, we consider a variation of the WMFVS method that pays more attention to the total weight of an FVS than to its size; i.e., it finds the maximum-weighted FVS. We call this method WFVS. In the next sections, we can see that WMFVS has a higher precision than WFVS, while WFVS has an advantage in recall.

## Results

### Data sets

In this study, we used the directed human protein interaction network [20] for the analyses; it contains 6338 genes (vertices) and 34814 directed interactions (arcs). To evaluate the relative prediction accuracies for cancer genes between our methods and existing methods, we collected cancer-related gene sets from five public databases: ONGene [21], TSGene [22], CGC [23], NCG [24] and MSigDB C6 [25]. Since not all genes from the data sets are contained in the directed human protein interaction network, we filtered the common genes in both a certain data set and the network. The sizes of these data sets are shown in Table 1.

**Table 1:** Size of each data set and the number of genes contained in the network (common genes)

	ONGene	TSGene	CGC	NCG	MSigDB
Number of genes	803	1217	723	2372	10,962
Common genes	490	641	525	1210	4184

In the rest of this paper, when we calculate the recall of various methods, we consider only the size of the common gene sets.

### Weight definition

To define the weights of genes, we first downloaded the RNA-seq data from TCGA [26], which contains gene expression data from 1102 breast tumour samples and 113 normal samples. Next, the counts of level 3 RNASeqV2 data were processed and transformed before being used for further analysis [27]. Specifically, we used the fold change (FC) value (with the binary logarithm and absolute value) between tumour and normal samples as the weight of each vertex (gene). For a specific gene *v*, its weight is calculated by the following formula:

1 $$\begin{aligned} v.w=\left| \frac{\Sigma _{i=1}^n \log _2 T_i}{n}-\frac{\Sigma _{j=1}^m \log _2 N_j}{m}\right| \end{aligned}$$

where $T_i$ is the expression value of tumour sample *i*, $N_j$ is the expression value of a normal sample *j*, and *n* and *m* are the numbers of tumour and normal samples, respectively. Intuitively, a high FC value corresponds to a high possibility of a cancer gene. Thus, it is reasonable to use the FC values as the weights of genes.

For the genes that appear in the network but have no expression values in the TCGA data (only 143 genes, 2.3% of the network size; these are called weight-loss genes), we gave them default weights of 0 rather than ignoring them; thus, if such a gene is essential at the topological level, it has the potential to be selected as a cancer gene, which may counteract the disadvantage of the traditional differential expression-based methods in dark gene-revealing and missing-data situations. Finally, all 6338 genes in the graph were weighted. The topological structure of the graph remained the same as in the original protein interaction network.

### Experiments and evaluation

**Fig. 1:**
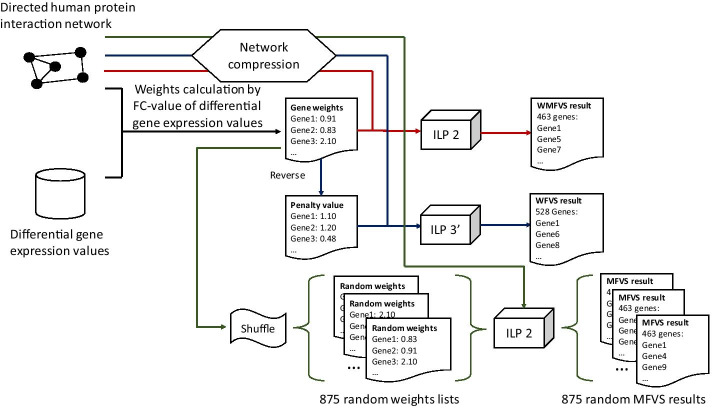
The experiment flowchart. The red, blue and green lines correspond to the WMFVS, WFVS and random MFVS pipelines, respectively

The whole experiment process is shown in Fig. 1.

First, we analysed the directed human protein interaction network with traditional MFVSs and obtained a set of 463 vertices. Then, we used our WMFVS method on the same network (the weights were derived from the FC values). We also used the inverses of the weights as the penalty values and applied them to our WFVS method.

Because of the non-uniqueness of the MFVS method, it is not a general evaluation if we consider only one MFVS result. Therefore, we calculated a set of random MFVSs by applying the WMFVS method with randomly shuffled gene weights. First, we planned to compute 1000 random MFVSs for analysis. However, since the Gurobi optimizer (version 8.1.0) does not always output a real optimal solution (e.g., even when we restrict the size of the output to be exactly 463, which is the size of the MFVS, sometimes the sizes of the output are smaller than 463), we filtered the obviously incorrect results and verified all the other outputs as MFVSs. Finally, we obtained 875 approved random MFVSs (since some MFVSs may be lost in the *ignore_w* operation and the MFVSs are not distributed uniformly, not all possible MFVSs have the same possibility of random selection).

The WMFVS and WFVS result data can be found in the supplementary data. The random MFVS data are placed in https://github.com/lrming1993/WMFVS_codes.

To evaluate the results of these three methods, we first checked the graph-level results (see Table 2).

**Table 2:** The graph-level results of each method

	Output size	Run time (s)	Sum weight	Average weight of each vertex
MFVS	463	4.0	319.5	0.69
WMFVS	463	35.9	379.3	0.82
WFVS	528	23.6	496.4	0.94

The run time of MFVS is due to the use of the traditional non-weighted MFVS method. The sum weight of MFVS uses the average value from 875 randomly weighted WMFVSs.

As we expected, the WMFVS method obtained a better total weight than the traditional MFVS. However, the result of WMFVS is not always better than that of MFVS. The total weight of the output of the traditional MFVS method is random (the output is related to the graph structure but has no relevance to the vertex weights), so it is possible for MFVS to output a highly weighted vertex set, even higher than the weight of the calculated WMFVS (Gurobi may not always give a real optimal result because of its numerical instability). However, our WMFVS method clearly has better stability.

The WFVS method returned an FVS with 528 vertices, which is approximately 14% larger than the size of the MFVS. The selected WFVS has a better average weight than both the MFVS and WMFVS. This result is consistent with our purpose for WFVSs, which focuses on the total weight rather than the size of the FVS.

**Fig. 2:**
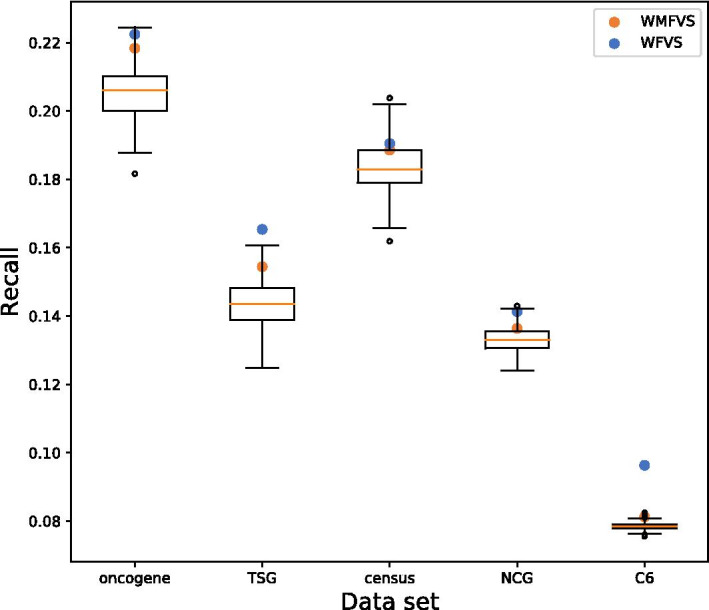
Distributions of the recalls for the random MFVSs (boxplot), the WMFVS method (orange circle), and the WFVS method (cyan circle) on different cancer gene data sets

Then, we used the five prepared cancer-related gene data sets to evaluate the results of these three methods. We verified the recall of the three FVS methods in the five data sets. The results are shown in Table 3 and Fig. 2.

**Table 3:** The recall of each method in different gene sets

	ONGene	TSGene	CGC	NCG	MSigDB
MFVS (average)	20.6%	14.4%	13.3%	13.2%	7.9%
WMFVS	21.8%	15.4%	18.8%	13.6%	8.1%
WFVS	22.2%	16.5%	19.0%	14.1%	9.6%

We can see that WMFVS and WFVS have better recall than traditional MFVS in all five sets, which is a benefit of the well-defined gene weights (especially for WFVS). Furthermore, we calculated the p-values of WMFVS and WFVS for 875 random MFVSs (Table 4).

**Table 4:** The p-values of WMFVS and WFVS for random MFVSs

	ONGene	TSGene	CGC	NCG	MSigDB
WMFVS	0.0491	0.0434	0.2537	0.2011	0.0069
WFVS	0.0069	0.0	0.1771	0.0091	0.0

For a certain data set, denote the recall of WMFVS by $R_{WMFVS}$. The recalls of all random MFVSs compose a set $R_{random}$. Then the p-value of WMFVS is calculated by the following formula:

2 $$\begin{aligned} p_{WMFVS}=\frac{|\{R|R \ge R_{WMFVS}, R \in R_{random}\}|}{|R_{random}|} \end{aligned}$$

The calculation of the p-value of WFVS is the same as above.

Next, as control methods, we considered several other kinds of methods of cancer gene prediction.

1. Randomly select 463 genes (select 100 times and take the average performance).
2. Select the 463 highest-weighted genes, which is a traditional differential expression-based method.
3. Select the set of genes that appear in at least 49.5% MFVSs (we used 49.5% since the number of genes was exactly 463).

**Fig. 3:**
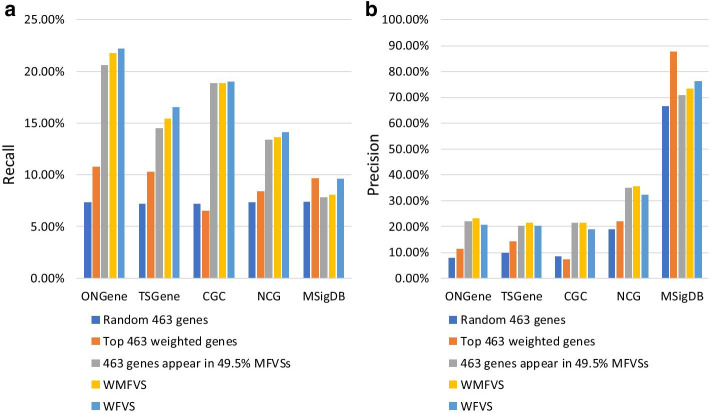
The recalls and precisions of all the methods

Method (2) uses only weights for classification (i.e., a pure differential expression analysis method), while method (3) uses only graph theoretic results (i.e., a pure network analysis method). Method (3) selects the most common genes that appear in the MFVS. Intuitively, these genes should have great significance in the graph topology. The recalls and precisions of all these methods are listed in Table 5. Additionally, see Fig. 3.

**Fig. 4:**
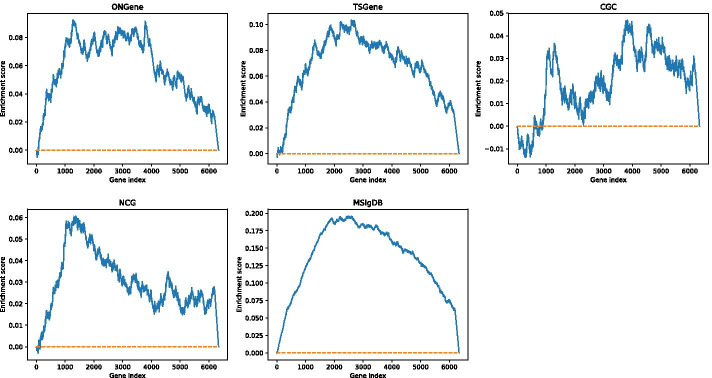
The enrichment score of each data set

**Table 5:** The recalls (and precisions) of all the methods

	463 random genes	Top 463 weighted genes	Genes appearing in 49.5% MFVSs	WMFVS (size: 463)	WFVS (size: 528)
ONGene	7.3% (7.8%)	10.8% (11.4%)	20.6% (21.8%)	21.8% (23.1%)	22.2% (20.6%)
TSGene	7.2% (9.9%)	10.3% (14.3%)	14.5% (20.1%)	15.4% (21.4%)	16.5% (20.1%)
CGC	7.2% (8.2%)	6.5% (7.3%)	18.9% (21.4%)	18.9% (21.4%)	19.0% (18.9%)
NCG	7.3% (19.0%)	8.4% (22.0%)	13.4% (35.0%)	13.6% (35.6%)	14.1% (32.4%)
MSigDB	7.4% (66.5%)	9.7% (87.5%)	7.8% (70.8%)	8.1% (73.4%)	9.6% (76.3%)

## Discussion

### Performance and enrichment score

In ONGene, TSGene and MSigDB, both WMFVS and WFVS have good p-values, but for CGC and NCG, the p-value is relatively high. One major reason is that there exists some correlation between the classification metric of the data set and the defined gene weight. To analyse this correlation, we utilized the enrichment score (ES) from GSEA [25], which reflects the degree to which a set *S* is overrepresented at the extremes (top or bottom) of an entire ranked list.

First, we sorted all the genes from the network by weight from high to low. Then, for a certain cancer gene set *S*, we traversed the sorted gene list, increasing a running-sum statistic when we encountered a gene in *S* and decreasing it when we encountered a gene not in *S*. We modified the increment and decrement value to ensure that the running sum was 0 at the end of the gene list. The enrichment scores of the five data sets are shown in Fig. 4.

It is easy to see that the ONGene, TSGene and MSigDB data sets are significantly enriched at the tops of the lists. Although NCG seems enriched at the top, its ES is relatively low; the ES of CGC is even worse than that of NCG. The best enriched data set is MSigDB. Since this data set was constructed directly from microarray gene expression data from cancer gene perturbations, it is closely related to differential expression values. The ES value explains the different performances of WMFVS and WFVS in different data sets.

Table 5 and Fig. 3 show that, except in MSigDB, WMFVS has the best precision and WFVS has the best recall. In MSigDB, cancer genes are closely related to the differential expression values of genes in breast cancer, leading to a precision of 87.5% for the simple weight-based method (i.e., method (2)). In this case, integration of the network structure may decrease the precision. However, in most cases, it is hard to find such a closely related metric for classification. We can observe that in other data sets, method (2) performs worse than the other methods. The results support the effectiveness of our WMFVS and WFVS methods.

### Dark genes

As mentioned previously, traditional differential expression-based methods are not able to find graph-level important genes that have low differential expression values, i.e., dark genes. In our research, we defined a dark gene as a gene that has a relatively low weight (i.e., a low differential expression value) but is recorded as a cancer gene in the cancer gene data base(s). Specifically, we first derived the differentially expressed genes (DEGs) by using the criteria of $|\log _2 FC|\ge 1$ and adjusted p-value $\le 0.05$ from the TCGA breast cancer RNA-seq data, where *FC* is the fold change value of a certain gene. Based on these criteria, we found 4245 DEGs (called the DEG set). Next, we curated the dark gene set from each cancer gene data set by excluding these DEGs.

**Fig. 5:**
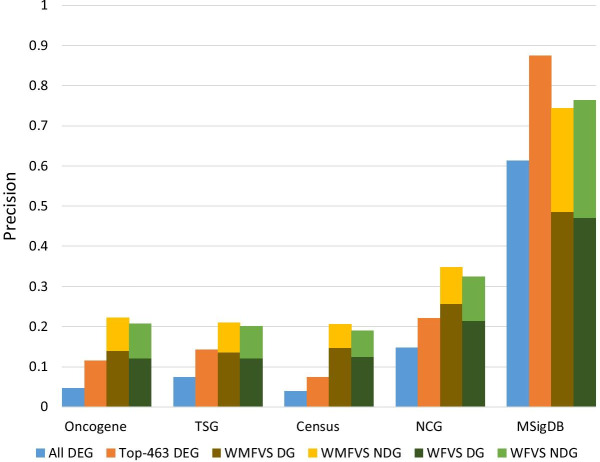
Comparison of the precision of the all-DEG set, top-463 DEG set, WMFVS set and WFVS set in five different cancer gene data sets. ‘DG’ and ‘NDG’ represent the ratios of dark genes and non-dark genes, respectively. Note that the all-DEG and the top-463 DEG sets contain no dark genes

In our experiments, we further selected the top 463 of the highest-weighted genes (i.e., the most differentially expressed genes; called the top-463 DEG set) to avoid an unbalanced gene number in comparison to the WMFVSs and WFVSs identified by the WMFVS and WFVS methods, respectively. For each of the cancer gene data sets, the precisions of the all-DEG set, top-463 DEG set, WMFVS and WFVS are shown in Fig. 5.

Figure 5 shows that our WMFVS and WFVS methods display better precision than the traditional DEG-based method (i.e., the all-DEG set and the top-463 DEG set) in four of five cancer gene data sets. Moreover, approximately 60–70% of the genes are dark genes, which were detected by using our WMFVS and WFVS methods but ignored by the traditional DEG method. Even for the MSigDB C6 data set, which was generated directly from microarray data or from internal unpublished profiling experiments involving the perturbation of known cancer genes, the WMFVS and WFVS methods also have a good ability to detect dark genes. In summary, our WMFVS and WFVS methods have an advantage in identifying dark genes that are hard to find by using traditional DEG methods.

### Missing-data cases

In this study, to retain the topological structure of the network, the weight-loss genes are assigned default weights of 0 rather than being removed. By further analysis, we found 3 weight-loss genes (i.e., CDC2, ZBTB8 and TADA3L) included in the WMFVS result, 7 weight-loss genes (i.e., CDC2, ZBTB8, RhoGDI, TADA3L, RNF12, NP and MAP3K7IP1) contained in at least one of the 875 random MFVS results, and no weight-loss genes in the WFVS result. In particular, CDC2 and ZBTB8 were included in all the random MFVS results as well as in the WMFVS result. The CDC2 gene is related to the highly conserved protein CDK1, which functions as a serine/threonine kinase and is a key player in cell cycle regulation [28]. The CDC2 gene is also considered a cancer-related gene whose overexpression may play an important role in human breast carcinogenesis [29]. While little is known about the ZBTB8 gene, the same ZBTB family protein, ZBTB7A, has been implicated in high expression in cancer tissue and the breast cancer cell lines MDA-MB-231 and MCF-7 [30], suggesting that ZBTB8 may act as a transcriptional repressor or be involved in tumorigenesis. The uncovering of CDC2 and ZBTB8 genes illustrates that the WMFVS method may address the disadvantage of traditional DEG methods in missing-data cases.

## Conclusion

We present several new methods for cancer gene prediction. Our WMFVS method uses differential gene expression to select MFVSs, improving the stability of the general MFVS algorithm and obtaining a much better result than the differential gene expression-based method when the weights of the genes are well defined. Our WFVS method is a variant of WMFVS, which aims at finding an FVS in the network that contains the maximum total weight. This method obtains better recall than WMFVS by sacrificing precision. Thus, generally, if the researcher wants to reveal as many potential cancer genes as possible, WFVS is better; if the researcher prefers better precision, then WMFVS is better. Furthermore, since WFVS ignores the restriction of the output size, it focuses more on the vertex weight than WMFVS. Therefore, if the researcher has good confidence in the weight definition, i.e., the weights are closely related to the classification, WFVS will have a better result than WMFVS. We can see this from the data analyses on the MsigDB data set, which has the highest enrichment score on our defined weights. However, in many cases, since we are not sure whether the defined weights are closely related to the classification, using WMFVS will maintain better precision for the prediction.

WMFVS and WFVS take advantage of both bio-data and the network structure. They can be useful in novel cancer gene prediction and evaluation, and the same idea may also be applied to other bioinformatics problems. The main challenge of our methods is the definition of the weights. WMFVS and WFVS can perform very well when the weights are well defined but may display limited performance when the weights are not directly related to the category. Another issue concerns graph compression. In our experiments, the traditional MFVS method analysed the compressed graph (with the *ignore* operation; see details in the next section), which contained 660 vertices and 5604 arcs, and it was efficient and took only approximately 4 seconds to obtain the result. The input graph of WMFVS and WFVS was compressed using the limited $ignore\_w$ operation (see details in the next section), which contained 2348 vertices and 17283 arcs. Because of the different input scales, WMFVS and WFVS were not as efficient as the simple MFVS method, although the time costs were still acceptable. The development of new algorithms for weighted graph compression is left as future work.

## Methods

### Graph compression

In biological networks, a network usually contains tens of thousands of vertices and hundreds of thousands of arcs. In many cases, processing a large network is not practical because of the NP-hardness of the MFVS problem [12]. Generally, we can compress the original graph to a simpler graph that maintains (or can restore) the size of the MFVS of the original graph.

In the following sections, we define *v*.*suc* and *v*.*pre* as the sets of successors and predecessors of vertex *v*, respectively. Let $v_i$ be a vertex in a network *S*. Consider the following three cases [18]:

1. $v_i \in v_i.suc$, i.e., $v_i$ has a self-loop; then, $v_i$ should be in all FVSs, otherwise the self-loop cannot be removed.
2. $v_i.suc=\emptyset$ (or $v_i.pre=\emptyset$); then, $v_i$ is not in any MFVS, since it is not in any cycle.
3. $|v_i.suc|=1$ (or $|v_i.pre|=1$); let $v_j$ be the only successor (or predecessor, respectively) of $v_i$; then, any cycle containing $v_i$ also contains $v_j$.

For C1, we use a temporary list $\Delta M$ to record $v_i$; we add $v_i$ to $\Delta M$ and remove $v_i$ and all its incoming and outgoing arcs from the graph. We use $remove(v_i)$ to denote this removing process.

For C2, since $v_i$ is not in any MFVS, we can safely use $remove(v_i)$ without any change to the possible MFVSs.

For C3, assume $v_i$ is in some cycle *c*. If we attempt to break *c* by removing $v_i$, then it is equally good (sometimes better) to remove $v_j$ rather than $v_i$. Here, we connect all predecessors of $v_i$ to all its successors and then use $remove(v_i)$. We denote this connecting and removing operation by $ignore(v_i,S)$, where *S* is the current graph to which $v_i$ belongs. The procedure is as follows:

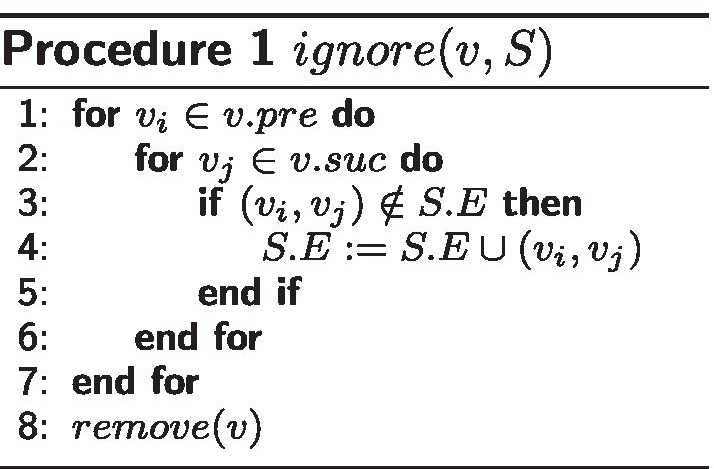



In the above procedure, *v* is a vertex in graph *S*, and *S*.*E* is the arc set of graph *S*. Then we have the following procedure to compress a graph *S*:

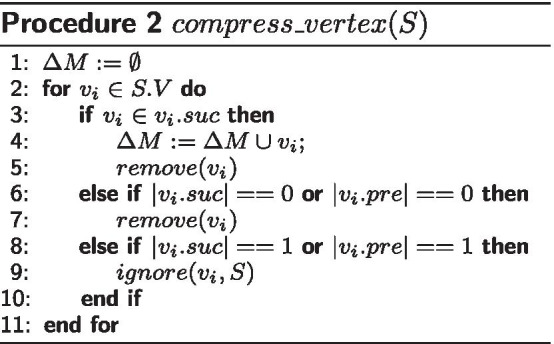



We repeat this procedure until *S* cannot be modified.

Furthermore, we use the strongly connected components (scc’s) [17, 19] to reduce the arcs. Since an arc between two scc’s is not in any cycle, the deletion of these arcs will not change any MFVSs. We use $compress\_scc(S)$ to denote the operation that removes all arcs between two different scc’s in *S*. The whole graph compressing procedure is as follows:

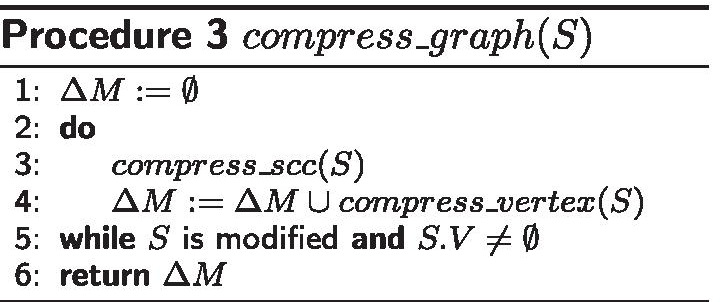



The returned $\Delta M$ contains the vertices that are always in any MFVS, and the union of $\Delta M$ and any MFVS of the compressed graph will be an MFVS of the original graph.

Note that not all MFVSs of the original graph can be obtained from the above method. Some MFVSs are lost in the *ignore* operation, while in a weighted MFVS problem, the lost MFVSs may have the maximum weight. For the weighted case, we modify the *ignore* operation to consider the weights of vertices (only for positive-weighted cases). The following method ensures that the maximum-weight MFVS (the WMFVS) will not be lost:

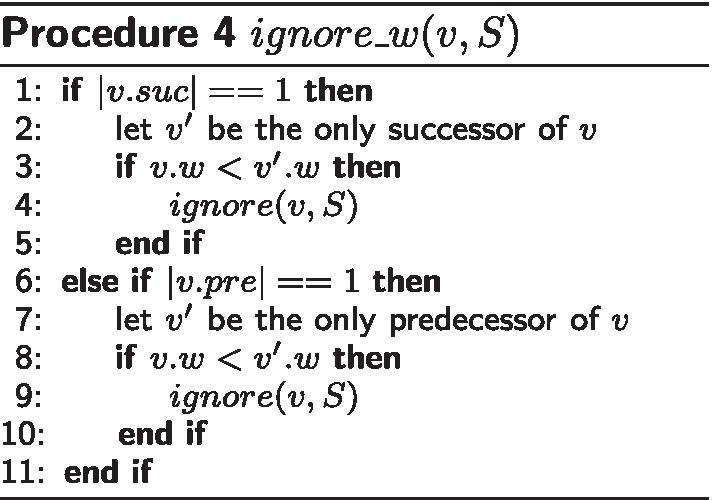



where *v*.*w* denotes the weight of vertex *v*.

#### Theorem 1

*When the weights of the vertices are positive, the vertices ignored in the* $ignore\_w$ *procedure are not in any WMFVS.*

#### Proof

Assume $v.pre=\{v'\}$, $v.w<v'.w$, and *v* belongs to a WMFVS *M*. Then, $v' \notin M$, otherwise $M':=M-\{v\}$ is still an FVS, which has fewer vertices than an MFVS.

Now consider $M'':=(M-\{v\}) \cup \{v'\}$. It is obvious that $M''$ is an MFVS. Since $v'.w>v.w$, we have $\Sigma _{v_i \in M} v_i.w< \Sigma _{v_j \in M''} v_j.w.$ Thus, *M* cannot be a WMFVS, i.e., if *v* has only one predecessor and the weight of *v* is less than that of the predecessor, then *v* does not belong to any WMFVS.

The proof is similar when *v* has only one successor and the weight of *v* is less than that of the successor. $\square$

### ILP formulation for MFVS and WMFVS

After the compressing procedure, if the compressed graph is not empty, we can use an ILP method [17] to solve the remaining MFVS problem. For each remaining vertex $v_i$, we add two parameters $x_i$ (Boolean) and $k_i$ (integer), where $x_i$ denotes whether $v_i$ is included in the output MFVS result and $k_i$ is a temporary parameter used in the ILP. The ILP formulation is as follows:

**ILP1:**

$$\begin{array}{*{20}c} \textbf{Minimize} &  \Sigma x_i \\ \textbf{Subject to} &  k_i-k_j+nx_i \ge 1 \: \forall (v_i,v_j)\in E  \\  & \textbf{where} \: 0 \le k_i \le n-1 \: \textbf{and} \: x_i \: \textbf{is Boolean} \end{array}$$

where *E* is the arc set of the remaining graph.

These constraints ensure that the selected vertices compose an FVS of *S*, while the objective function means that the selected FVS has a minimum size, i.e., it is an MFVS.

Now we consider the weighted case of the MFVS problem. Given a graph *S*, where each vertex $v_i \in S.V$ has a weight $v_i.w$ (in what follows, we use $w_i$ to denote $v_i.w$ if there is no ambiguity), the WMFVS problem is to find an MFVS of *S* that has the maximum total weight. Assuming we already know the size *s* of the MFVS (by ILP1 or some estimation method such as that of [31] or [32]), the following formulation optimizes the selected MFVS as a WMFVS:

**ILP2:**

$$\begin{array}{*{20}c} \textbf{Maximize} &  \Sigma w_i x_i  \\ \textbf{Subject to} &  \Sigma x_i=s \\ &  k_i-k_j+nx_i \ge 1 \: \forall (v_i,v_j)\in E  \\ &  \textbf{where} \: 0 \le k_i \le n-1 \: \textbf{and} \: x_i \: \textbf{is \,Boolean}  \end{array}$$

The constraint $\Sigma x_i =s$ ensures that the selected FVS is an MFVS, while the objective function selects the maximum-weight MFVS among all possible MFVSs.

### Maximum-weight FVS

**Fig. 6:**
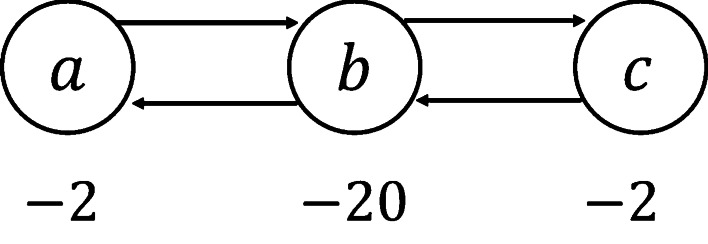
A simple example. In this case, the total weight may be more important than the size of an FVS

In the WMFVS problem, we first restrict the size of the FVS to be minimal and then select the maximum-weight MFVS as the objective. However, sometimes the weight may be more important than the size of an FVS. As an example, in Fig. 6, the WMFVS is $\{b\}$, which has a total weight of $-20$. If we do not restrict the minimum size of the set, the FVS $\{a,c\}$, which has weight $-4$, seems better.

Here we define a variant of the WMFVS problem, which ignores the exact size of the output vertex set, as follows: Given a graph *S*, where each vertex $v_i \in S.V$ has a weight $v_i.w$ (or $w_i$), the weighted FVS (WFVS) problem is to find an FVS of *S* that has the maximum total weight. We can simply use a similar ILP as ILP2 to solve the WFVS problem.

**ILP3:**

$$\begin{array}{*{20}c} \textbf{Maximize} &  \Sigma w_i x_i  \\ \textbf{Subject to} &  k_i-k_j+nx_i \ge 1 \: \forall (v_i,v_j)\in E  \\ &  \textbf{where} \: 0 \le k_i \le n-1 \: \textbf{and} \: x_i \: \textbf{is\, Boolean} \end{array}$$

However, simply removing the constraint $\Sigma x_i =s$ may lead to a trivial solution when the weights of the vertices are positive, since the set of all vertices will always be a WFVS. Here we consider two methods to avoid the trivial solution:

1. Modify all weights to be negative. Assume the maximum weight of the vertices is $w_m$; then, for each weight $w_i$, modify it to $w_i:=w_i-w_m-\epsilon$. Here, $\epsilon$ is a small positive constant to ensure that all weights are negative. The ILP is the same as ILP3.
2. Reverse the weights to penalty values. We can simply do this by taking the inverse of each $w_i$, i.e.,
  $$\begin{aligned} p_i=\left\{ \begin{array}{ll} \frac{1}{w_i} &{} \mathbf{if} \, w_i \ne 0 \\ \infty &{} \mathbf{if} \, w_i = 0 \end{array} \right. \end{aligned}$$
  Then, modify the ILP3 formula as follows: **ILP3’:**
  $$\begin{array}{*{20}c} \textbf{Minimize} &  \Sigma p_i x_i  \\ \textbf{Subject to} &  k_i-k_j+nx_i \ge 1 \: \forall (v_i,v_j)\in E\\ &  \textbf{where} \: 0 \le k_i \le n-1 \: \textbf{and} \: x_i \: \textbf{is \,Boolean} \end{array}$$

In our research, we examined both ways of calculating the weights in the WFVS method. We found that the first modification is more unstable when running the ILP process, i.e., more obviously wrong ILP results appeared. Thus, we chose to use the second method to compute the weights in the WFVS method; i.e., we reversed the weights to be penalty values, which are always positive values.

In the second method, we need to avoid the ‘division by zero’ error. To this end, we used the simple heuristic formula below.

Let *l* be a large number (in our program, we used 65536); then, the penalty is calculated by:

$$\begin{aligned} p_i=\left\{ \begin{array}{ll} \frac{1}{w_i} &{} \mathbf{if} \, w_i \ge \frac{1}{l} \\ l &{} \mathbf{if} \, w_i < \frac{1}{l} \end{array} \right. \end{aligned}$$

## Experimental environment

We implemented all the methods in Python 3.7.0 with an Intel(R) Core(TM) i7-7700 CPU and 32.0 GB RAM. The *compress_scc* procedure uses Gabow’s algorithm [33]. The ILP processing is based on Gurobi 8.1.0 [34].

## Supplementary information

**Additional file 1.** .The data of the edges of the compressed network; the *ignore* operation was used. This file was used for the traditional MFVS computation.

**Additional file 2.** The data of edges of the compressed network; neither *ignore* nor *ignore_w* was used. This file was used for WFVS computation.

**Additional file 3.** The data of the edges of the compressed network; the *ignore_w* procedure was used. This file was used for WMFVS computation.

**Additional file 4.** The weights of all genes in the network.

**Additional file 5.** The ∆*M* computed from procedure 3, where the *ignore* operation was used. This file is necessary when using Additional file 1 to compute the MFVS.

**Additional file 6.** The ∆*M* computed from procedure 3, where the *ignore_w* operation was used. This file is necessary when using Additional file 3 to compute the WMFVS.

**Additional file 7.** The result of the traditional MFVS method.

**Additional file 8.** The result of the WMFVS method.

**Additional file 9.** The result of the WFVS method.

## Data Availability

The source code is on GitHub (https://github.com/lrming1993/WMFVS_codes). The random MFVS results can also be found at this URL. All data generated during this study are included in this published article and its supplementary information files.

## Abbreviations

MFVS: Minimum feedback vertex set
WMFVS: Weighted minimum feedback vertex set
WFVS: Maximum weighted feedback vertex set
ILP: Integer linear programming
FC: Fold change
ES: Enrichment score
scc: Strongly connected component
DEG: Differentially expressed gene
